# Effect of CPAP Therapy on 24-Hour Intraocular Pressure-Related Pattern From Contact Lens Sensors in Obstructive Sleep Apnea Syndrome

**DOI:** 10.1167/tvst.10.4.10

**Published:** 2021-04-14

**Authors:** María Jesús Muniesa, Iván Benítez, Juan Ezpeleta, Manuel Sánchez de la Torre, Marta Pazos, Elena Millà, Ferrán Barbé

**Affiliations:** 1 Institut Clínic d'Oftalmologia, Hospital Clínic de Barcelona, Universitat de Barcelona, Barcelona, Spain; 2 Institut de Recerca Biomèdica de Lleida (IRBLleida)—Group of Translational Research in Respiratory Medicine, Lleida, Spain; 3 Centro de Investigación Biomédica en Red de Enfermedades Respiratorias (CIBERES), Madrid, Spain; 4 Ophthalmology Department, Arnau de Vilanova University Hospital, IRBLleida, Catalonia, Spain

**Keywords:** 24-hour intraocular pressure monitoring, sleep apnea syndrome, contact lens sensor, continuous positive airway pressure, CPAP, glaucoma, fluctuations intraocular pressure

## Abstract

**Purpose:**

To evaluate the effect of continuous positive airway pressure (CPAP) therapy on 24-hour intraocular pressure (IOP)-related pattern from contact lens sensors (CLS) in obstructive sleep apnea syndrome (OSAS).

**Methods:**

Prospective, observational, case series study. Twenty-two eyes of 22 newly diagnosed patients with severe OSAS were included. A first 24-hour CLS measurement was performed before CPAP therapy was started, and a second 24-hour CLS monitoring was performed after beginning CPAP. We analyzed the amplitude and the maximum and minimum IOP-related values (m Veq). We also analyzed IOP-related measurements at five-minute intervals throughout the first hour of nocturnal acrophase, starting from when the patient fell asleep.

**Results:**

The baseline measurements showed significant fluctuations in the IOP, with the highest IOP readings being recorded at night (nocturnal acrophase) in 17 of 22 patients (77.27%). Nocturnal acrophase began when the patients laid down to sleep. During CPAP therapy, the patients showed a more marked increase in IOP in the initial phase of nocturnal acrophase, with significant differences at 20, 25, 30, and 55 minutes (*P* < 0.05).

**Conclusions:**

Most of patients with severe OSAS exhibited a nocturnal acrophase and the highest IOP readings at night. CPAP was associated with additional increase in IOP-related pattern for at least the first hour of CPAP use.

**Translational Relevance:**

Our results suggest that CPAP was associated with additional increase in IOP during the initial phase of nocturnal acrophase. This effect could be important in the management of patients with OSAS and glaucomatous progression.

## Introduction

Obstructive sleep apnea syndrome (OSAS) is characterized by recurrent, complete or partial, upper airway obstructions during sleep, and it is associated with a high incidence of cardiovascular and neurovascular disease.^1^ Continuous positive airway pressure (CPAP), which is the first-line therapy for OSAS, suppresses abnormal respiratory events, restoring sleep quality, and partly or completely reversing acute and chronic cardiovascular modifications associated with the disease.^2^ OSAS has also been linked to various ocular disorders, including floppy eyelid syndrome,^3^ keratoconus,^4^ nonarteritic anterior ischemic optic neuropathy,^5^ papilledema caused by increased intracranial pressure,^6^ and glaucoma.^7^^–^^11^ The relationship between OSAS and glaucoma remains incompletely understood, especially with regard to changes in intraocular pressure (IOP) in OSAS patients. The elevated IOP is recognized as the only modifiable risk factor for the development and progression of glaucoma.^12^ A positive correlation has been reported between IOP levels and the severity of OSAS.^13^ It has been published that on OSAS patients receiving different treatments, only the CPAP treatment could not decrease the risk of glaucoma.^14^ A number of studies have explored the effect of CPAP therapy on IOP in OSA patients, but with controversial outcomes,^15^^–^^19^ so the debate about whether CPAP is associated with an increase in IOP and whether therefore it is safe for OSAS patients with glaucoma persists.

The dynamic nature of IOP underlies the need for a clinical tool that would allow its continuous assessment over a 24-hour period and especially during the night, when the IOP often tends to increase. A major breakthrough was made when Leonardi and associates^20^ updated the concept of a soft contact lens with an embedded wireless sensor and developed an approved commercial product that records IOP-related patterns with no requirement of waking subjects up during the nocturnal sleep period.

The purpose of the present study was to compare 24-hour IOP-related patterns with contact lens sensors (CLS) in patients with OSAS in baseline situations and with CPAP therapy. We hypothesized that 24-hour pressure-related pattern assessments could provide better information for the IOP in OSAS patients and CPAP effect.

## Material and Methods

The present study was a prospective, observational case series study performed at the Arnau de Vilanova-Santa María University Hospital, Lleida, Catalonia, Spain, after approval by the Ethics Committee of the Arnau de Vilanova University Hospital (CEIC-1431). It followed the tenets of the Declaration of Helsinki (1964), and written informed consent was obtained from each subject.

### Study Subjects

Twenty-two patients with newly diagnosed OSAS were included between February 2017 and September 2018. One eye of each patient was included in the study. All of these patients had severe OSAS, and CPAP treatment was indicated.

The patients underwent a comprehensive ophthalmic examination, which included visual acuity, with a recording of refractive correction; slit-lamp and fundoscopy examinations; Goldmann applanation tonometry; ultrasonic corneal pachymetry (Ocuscan RXP; Alcon Laboratories, Inc, Geneva, Switzerland); visual field analysis with a Humphrey Field Analyzer (Carl Zeiss Meditec, Dublin, CA, USA; SITA-standard program, central 24-2 threshold test); and measurement of the thickness of the retinal fiber layer, using optical coherence tomography (Cirrus OCT; Carl Zeiss Ophthalmic Systems Inc, Dublin, CA, USA). The patients were all over 18 years old. The exclusion criteria were a corneal radius outside the manufacturer's recommended range of 40 to 48 D and taking medications that are known to have potential effects on IOP, such as local or systemic steroids. No participants had any history of eye disease, such as glaucoma or ocular hypertension, previous intraocular laser treatment, or ocular surgery, except for cataract surgery at least six months before enrollment. All included patients had IOP ≤ 21 mm Hg without treatment, normal visual field, normal optic disc and retinal nerve fiber layer, and open anterior chamber angle.

### Sleep Studies

Diagnosis of OSAS was reached on the basis of either conventional polysomnography (PSG) or a cardiorespiratory sleep study.^21^ Apnea was defined as an absence of airflow for at least 10 seconds, and hypopnea was defined as a clear (50%) reduction in airflow for at least 10 seconds, with a corresponding fall in oxygen saturation of at least 4%, or an arousal. OSAS were defined as the absence of airflow in the presence of chest or abdominal-wall motion. The apnea-hypopnea index (AHI) was calculated according to the average number of episodes of apnea plus hypopnea per hour of sleep or recording time. OSAS was excluded when AHI < 10. OSAS severity was scored as mild when the AHI was between 10 and 20, moderate when it was between 20 and 30, and severe when AHI > 30. Standard CPAP was indicated in all of the patients with OSAS included in the study. The air pressure of the CPAP that was assessed was the pressure at which most (if not all) apneas and hypopneas were prevented; it was measured in centimeters of water (cm H_2_O).

### Measurements With the Contact Lens Sensor

The 24-hour IOP-related pattern was monitored using a CLS (Sensimed Triggerfish; Sensimed AG, Lausanne, Switzerland). For all 22 patients included, we did a first monitoring of IOP with CLS before starting the CPAP therapy. A random eye assignment from card shuffling was used to determine the eye for CLS placement in each patient. For 16 of these 22 patients, we did a second monitoring with CLS during the CPAP treatment after they had started CPAP therapy one month before the second monitoring. The reasons for not performing CLS monitoring with CPAP in six of the patients were poor compliance with CPAP treatment (four patients) and the fact that they did not agree to a second monitoring (two patients).

The baseline IOP with Goldmann applanation tonometry was obtained by averaging the last three readings taken before starting the measurements with the CLS. The doctors who had been trained in CLS measurement (M.J.M., J.E.) fitted and removed each subject's CLS at the hospital. All monitoring started at between 1 p.m. and 3 p.m. The CLS is a highly oxygen-permeable soft contact lens whose key elements are two sensing-resistive strain gauges that are capable of registering circumferential changes in the area of the corneoscleral junction. The device is based on an approach to registering IOP patterns in which changes in corneal curvature and, hence, ocular volume are assumed to be related to changes in IOP.^22^ The CLS recorded a relative IOP-related signal (rather than the absolute IOP). The measurement unit of the CLS is the millivolt equivalent (mV eq); this is unique to the Sensimed Triggerfish. The median values were monitored for 30 seconds, once every five minutes, thereby providing 288 points over a 24-hour period. The most appropriate size for the contact lens was determined using keratometry readings. The potential risks associated with the use of the lens were discussed with each patient. They included discomfort, dry eyes, blurred vision, foreign body sensation, itching, swelling, and irritation. A local anesthetic (oxybuprocaine hydrochloride 0.4%) was applied to improve comfort when fitting the lens. Then, after its placement, its positioning was checked using a slit lamp. All patients were instructed to return 24 hours later for the removal of the lens and data collection. The subjects were instructed to record the time at which they went to bed and woke up. They did not have any restrictions on their posture during the monitoring process. After the removal of the CLS, the slit-lamp examination was repeated.

The second 24-hour monitoring with CLS was carried out after beginning CPAP treatment for one month, with patients wearing their masks during IOP measurements. Compliance with CPAP was considered acceptable if the device was used for at least four hours per night.

### Analysis of CLS Measurements

After 24 hours, the CLS was removed and the data were downloaded and analyzed using proprietary software incorporating cosinor-based rhythmometry; this is a function that is commonly used to study circadian biological rhythms. The use of modified cosinor rhythmometry for the analysis of 24-hour IOP-related patterns obtained with the CLS^23^ simplified data interpretation by providing several key parameters related to the circadian IOP rhythm: acrophase and bathyphase (the timing of the CLS signal peaks and troughs) and also signal amplitude expressed as an indicator of fluctuations in IOP.^24^ The software also automatically identified the sleeping period by analyzing blink patterns, with blinks (which produce a transient spike in the electrical signal) being less frequent during sleep.^25^ This was then cross-checked with the self-reported sleep time provided by each subject.

The night-time, or nocturnal, period was defined as the time from when the patient went to sleep until they woke up. The curves obtained from monitoring were classified into three circadian IOP-related pattern groups: nocturnal pattern, diurnal pattern, and absence of pattern. In the nocturnal pattern, the nocturnal acrophase was higher than the diurnal pattern, with the maximum value being nocturnal. In the diurnal pattern, the diurnal acrophase was higher than the nocturnal pattern, with the maximum value being diurnal. In the group without any pattern, the average diurnal amplitude was similar to the average nocturnal amplitude without an acrophase period.

We also determined the increase in the IOP during the early phase of nocturnal acrophase by measuring the IOP-related pattern over the period from when the patient went to sleep until one hour later. We compared this parameter in patients both before starting CPAP treatment and during CPAP use. To compensate for the possible effect of the supine position on IOP, we only included in this analysis those patients who started using CPAP at the same time that they went to sleep (11 of 16 patients with a second monitoring during CPAP). So we included 11 eyes of 11 patients for this analysis. The increase in IOP during the early phase of nocturnal acrophase was measured using two different strategies. In the first strategy, two doctors examined the graphs to reach a consensus on the presence of a greater increase in IOP during the first hour of nocturnal acrophase; this was reflected by a steeper slope on the graph. The second strategy was based on measuring IOP-related differences at five-minute intervals during the first hour of nocturnal acrophase, both with and without CPAP treatment.

The doctors who had been trained in CLS measurement (M.J.M., J.E.) independently evaluated the curves for each patient to define the circadian IOP patterns and the increase in the IOP during the early phase of nocturnal acrophase in a masked, side-by-side comparison that was confirmed by an independent reader (I.B.).

### Statistical Analyses

Quantitative variables were described using the mean and standard deviation or median (interquartile range) according to the normality of the data. Absolute and relative frequencies were used to describe qualitative variables. We compared IOP measures (both with and without CPAP) by periods. Either the *t*-test or the Wilcoxon rank sum test (both for paired samples) was used to compare the quantitative variables and the χ^2^ test for qualitative variables. The nyctohemeral rhythm of IOP was fitted using the cosinor model. The characteristics of the circadian rhythm were expressed as the bathyphase (the time at which the lowest value encountered in the cycle occurred) and the acrophase (the time at which the highest value encountered in the cycle occurred). Two conditions were required to determine whether the rhythm was sinusoidal-like or if there was no rhythm: a significant *F*-ratio test of the global cosinor model and a rhythm percentage >0.7 (the coefficient of determination obtained by squaring the correlation between the observed and estimated values). To calculate the population-mean cosinor, single cosinors were first performed on each subject, and then the linearized parameters were averaged.^26^ *P* < 0.05 was considered statistically significant. All the statistical analyses and data processing procedures were performed using R software, version 3.5.2 (R software, Vienna, Austria).

## Results

### Baseline Characteristics

The study included an analysis of 22 eyes of 22 patients with newly diagnosed OSAS. The median (interquartile range) age of the patients was 66.5 (12.8–18.0), and most of the patients were men. All of the patients were obese (body mass index ≥ 30 Kg/m^2^) and had severe OSAS. Ophthalmic exploration gave normal results for all of the patients. A mean baseline IOP of 16 mm Hg was registered before sensor placement, with IOP values that were considered to lie within a normal range. The patient characteristics are detailed in Table 1.

**Table 1.** Characteristics of the Patients Included in the Study on the Basis of Reported Clinical Findings

	Cohort (n = 22)
Sociodemographic data	
Age (y), median (IQR)	66.5 [53.8;71.8]
Male gender, n (%)	18 (81.8%)
BMI (kg/m^2^), median (IQR)	34.2 [31.2;36.8]
Comorbidities	
Type II diabetes mellitus, n (%)	4 (18.1%)
Arterial hypertension, n (%)	10 (45%)
Polysomnographic parameters	
AHI, median (IQR)	52.5 [37.8;63.8]
Time with SaO_2_ < 90%, median (IQR)	36.0 [14.0;62.0]
Ocular data	
Study eye (right), n (%)	10 (45.5%)
IOP mm Hg baseline, median (IQR)	16.0 [12.8;18.0]
CCT (µm), median (IQR)	553 [531;579]
Vertical cup-to-disc ratio, median (IQR)	0.20 [0.14;0.21]

Data are expressed as means (SD) or numbers (%).

IQR, interquartile range; BMI, body mass index; AHI, apnea–hypopnea/h index; IOP, intraocular pressure; CCT, central corneal thickness.

### Baseline 24-Hour IOP-Related Patterns

**Figure 1.**
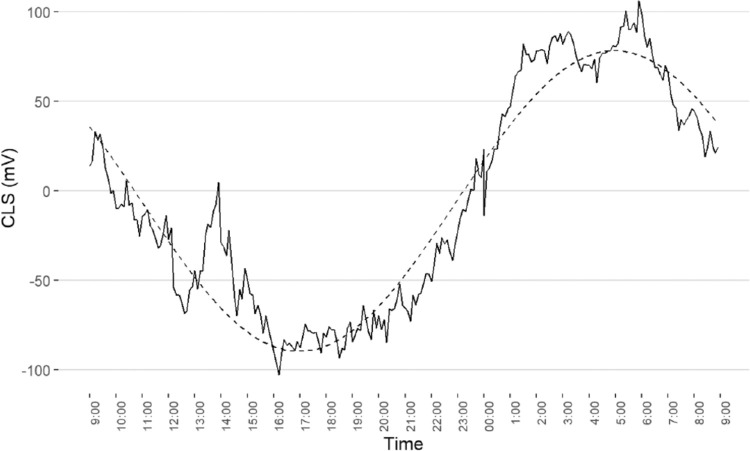
Population pattern of the circadian rhythm before continuous positive airway pressure treatment in severe OSAS patients (n = 22). CLS, contact lens sensor measures in m V eq. An acrophase was observed at 5:03 a.m.

**Figure 2.**
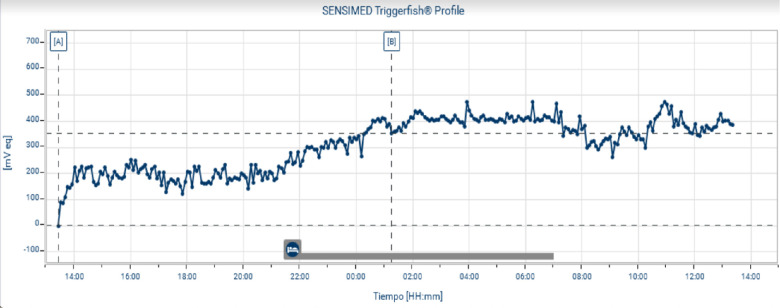
Twenty-four-hour intraocular pressure (IOP) monitoring with a contact lens sensor. This example was obtained from the monitoring of the IOP over 24 hours, as measured using a contact lens sensor. The patient's sleep period is indicated at the bottom. This patient showed a significant increase in nocturnal IOP associated with a nocturnal acrophase while patients were in bed compared with their result during daytime. We observed an increase in IOP which started when a patient lay on the bed.

The CLS accurately identified the sleeping periods when these were compared to the self-reported diary entries. The baseline measurements with CLS for the 22 patients before the use of CPAP are shown in Table 2. The population model of the IOP-related fluctuations in severe OSAS estimated a circadian rhythm with acrophase at 5:03 a.m., that is, a nocturnal acrophase (Fig. 1). In the individualized analysis, we observed a significant nyctohemeral fluctuation with a nocturnal pattern in 17 of the 22 patients studied (77.27%). We observed an increase in IOP associated with the moment when each individual initially lay down on the bed (Fig. 2). However, five of the 22 patients did not show any pattern and none of the patients exhibited a diurnal pattern.

**Table 2.** Baseline Measurements With CLS for Patients Before the Use of CPAP

	Cohort (n = 22), Mean (SD)
Amplitude, mV eq	114 (40.3)
Acrophase mV eq	218 (151)
Bathyphase mV eq	14.2 (155)
Acrophase time (a.m.)	6:27 (4:36)
Bathyphase time (p.m.)	5:48 (3:19)
Night acrophase pattern	17/22 (77.27%)

CLS, contact lens sensors; CPAP, continuous positive airway pressure.

### Comparison of IOP-Related Patterns With and Without CPAP

**Figure 3.**
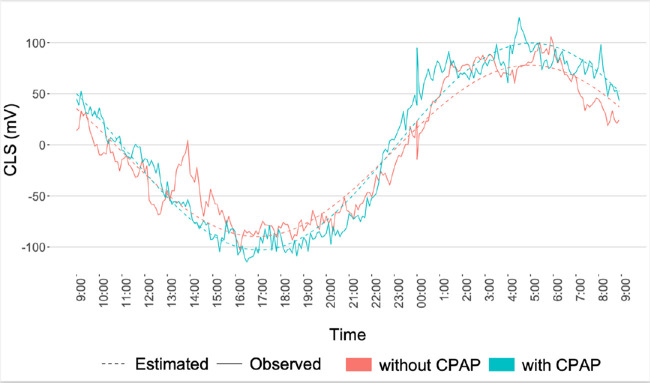
Population pattern of circadian rhythms before and during continuous positive airway pressure (CPAP) treatment in patients with severe obstructive sleep apnea syndrome. The use of CPAP did not modify the circadian pattern. It was showed the presence of nocturnal patterns in patients both before starting CPAP therapy and during CPAP. CLS, contact lens sensor measures in m Veq.

In 16 of the 22 patients who underwent pressure monitoring with CLS before starting the treatment with CPAP, a second monitoring was performed during CPAP use. The mean (SD) CPAP pressure required for patients was 11.6 ± 1.27 cm H_2_O. The measurements with CLS and the presence of nocturnal patterns in patients both before starting CPAP therapy and during CPAP use are shown in Table 3. There were no significant differences in such parameters as amplitude and the maximum and minimum IOP-related values. The CLS values measured during sleep time were significantly greater than those registered when the patients were awake; this was true both for patients without CPAP (209 ± 115 vs. 52.8 ± 84.1, *P* < 0.001) and those with CPAP (202 ± 170 vs. 73.7 ± 120, *P* < .001). During the second CLS monitoring session, three of the five patients (60%) who did not present a nocturnal pattern before starting CPAP therapy registered a circadian rhythm with a nocturnal acrophase. The population model for IOP-related fluctuations in severe OSAS patients with CPAP, as compared to those without CPAP, showed that CPAP did not modify the circadian pattern (Fig. 3).

**Table 3.** Measurements of Patients Using CLS, Conducted Both Before and After the Use of CPAP

	Period, Mean (SD)		
	Without CPAP (n = 16)	With CPAP (n = 16)	*P* Value
Amplitude, mV eq	109.53 (43.27)	110.95 (42.09)	0.882
Acrophase mV eq	199.75 (129.63)	187.59 (104.5)	0.687
Bathyphase mV eq	10.16 (134.06)	3.74 (120.11)	0.907
Acrophase time (a.m.)	6:39 (5.23)	5:32 (2.31)	0.887
Bathyphase time (p.m.)	5:37 (3.49)	5:00 (2.29)	0.597
Nocturnal pattern	11 (68.75%)	14 (87.5%)	0.392
mV eq – mean- awake	52.8 (84.1)	73.7 (120)	0.844
mV eq – mean- sleeping	209 (115)	202 (170)	0.688
mV eq – mean- 24h	128 (87.3–175)	152 (67.4–226)	0.945

CLS, contact lens sensors; CPAP, continuous positive airway pressure.

### The Increase in the IOP During the Early Phase of Nocturnal Acrophase

**Figure 4.**
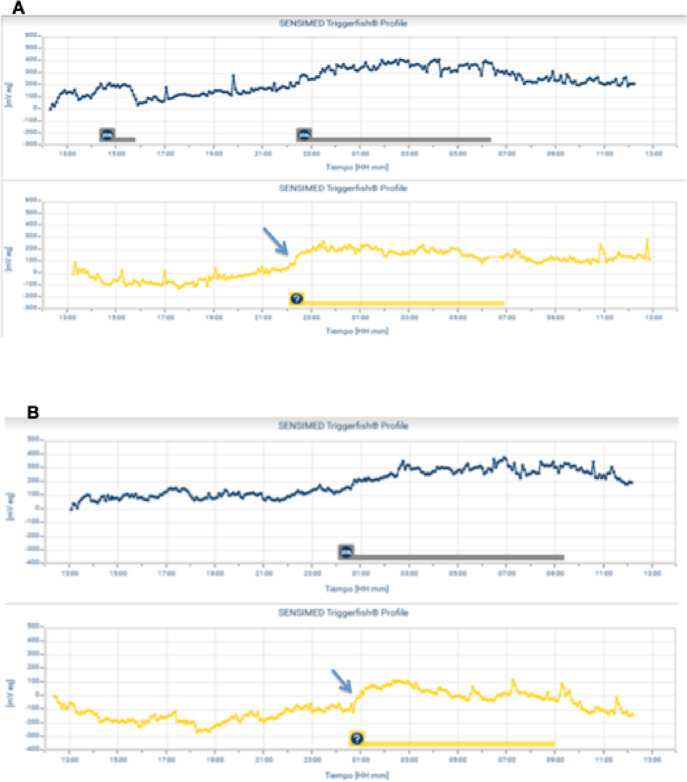
Two examples (A and B) of two patients for whom we compared the early phase of nocturnal acrophase both without (top graph) and with (lower graph) continuous positive airway pressure (CPAP), based on individualized analyses. A marked increase in nocturnal acrophase was observed after starting CPAP use (*arrow*). The patient's sleep period is indicated at the bottom of each graph.

**Figure 5.**
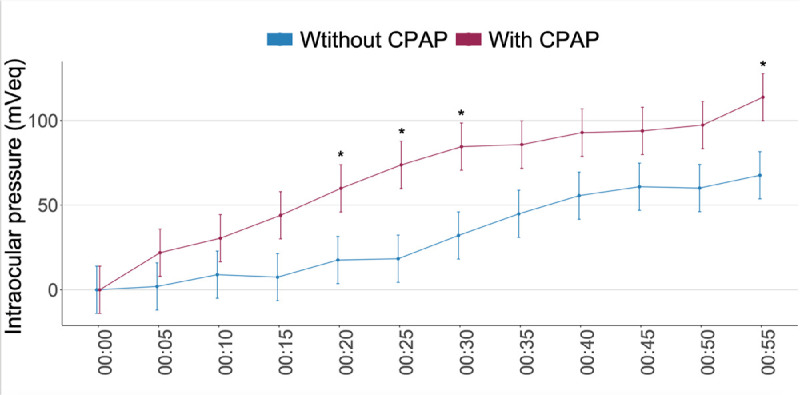
Intraocular pressure (IOP)–related differences at five-minute intervals during the first hour of nocturnal acrophase, both with and without continuous positive airway pressure (CPAP) therapy. Note: statistically significant differences (*P* < 0.05) are indicated with an *asterisk* in the graph. It was observed that the increase in IOP during the initiation of the nocturnal acrophase was faster with than without CPAP, with significant differences at 20, 25, 30, and 55 minutes.

We detected a steeper slope of the curve in the first hour of nocturnal acrophase during CPAP use in nine of 11 patients (81.8%) who started using CPAP at the same time that they went to sleep. They showed a relevant increase in the steepness of the slope of the curve. This result indicated a greater and faster increase in the IOP-related pattern associated with the initiation of CPAP use (Fig. 4). When we measured IOP-related differences at five-minute intervals during the first hour of nocturnal acrophase from the moment that the patient lay down to sleep, both with and without CPAP therapy, we observed an increase in the IOP-related pattern during the period with CPAP therapy; there were significant differences at 20, 25, 30, and 55 minutes (*P* < 0.05) (Fig. 5 and Table 4).

**Table 4.** IOP-Related Differences at Five-Minute Intervals During the First Hour of Nocturnal Acrophase, Both With and Without CPAP Therapy

			Difference	
Time (Min)	Without CPAP Mean (95% CI)	CPAP Mean (95% CI)	Mean (95% CI)	*P* Value
0:05	2 (−12 to 15.9)	21.9 (8 to 35.9)	−20 (−35.4 to −4.5)	0.361
0:10	8.9 (−5 to 22.9)	30.5 (16.5 to 44.4)	−21.6 (−37 to −6.1)	0.325
0:15	7.5 (−6.5 to 21.4)	44 (30.1 to 58)	−36.5 (−52 to −21.1)	0.091
0:20	17.6 (3.7 to 31.5)	59.9 (46 to 73.9)	−42.3 (−57.8 to −26.9)	0.047
0:25	18.3 (4.4 to 32.3)	73.8 (59.8 to 87.7)	−55.4 (−70.9 to −40)	0.011
0:30	32.1 (18.2 to 46)	84.6 (70.6 to 98.5)	−52.5 (−67.9 to −37)	0.012
0:35	44.9 (30.9 to 58.8)	85.8 (71.8 to 99.7)	−40.9 (−56.3 to −25.5)	0.062
0:40	55.6 (41.7 to 69.6)	92.9 (78.9 to 106.8)	−37.3 (−52.7 to −21.8)	0.089
0:45	60.9 (46.9 to 74.8)	93.8 (79.9 to 107.8)	−33 (−48.4 to −17.5)	0.136
0:50	60.1 (46.1 to 74)	97.3 (83.4 to 111.2)	−37.2 (−52.7 to −21.8)	0.089
0:55	67.6 (53.7 to 81.6)	113.8 (99.8 to 127.7)	−46.1 (−61.6 to −30.7)	0.003

The values are least squares mean estimated using a mixed model. There were significant differences at 20, 25, 30, and 55 minutes (*P* < 0.05).

IOP, intraocular pressure; CPAP, continuous positive airway pressure; CI, confidence interval.

## Discussion

The present study investigated 24-hour IOP-related patterns comparing OSAS patients, both with and without CPAP therapy. The data showed that (I) most of the severe OSAS patients exhibited a nocturnal acrophase pattern with higher CLS readings in the nocturnal period; and (II) CPAP therapy was associated with a significant increase in IOP-related patterns during the initial phase of nocturnal acrophase and particularly during the first 30 minutes after starting CPAP use, with this then remaining significant for at least one hour. To the best of our knowledge, the present study represents the first analysis of IOP-related fluctuations in patients with OSAS at baseline and during CPAP therapy carried out under physiological conditions and using a CLS.

We observed that the IOP-related increase in the nocturnal acrophase started when the individual lay down on the bed. This nocturnal IOP increase, also described in healthy patients, was probably related to the supine position and the corresponding increase in episcleral venous pressure.^27^

We observed that almost 23% of patients with severe OSAS exhibited an absence of rhythm without a nocturnal acrophase. There are many different factors that could explain an abnormal IOP pattern in OSAS, including the interruption of normal sleep cycles in patients with severe OSAS, circulating hormones while asleep, ocular perfusion pressure, and outflow facility in the sleeping position.^8^ These potential factors are disturbed by OSAS and normalized when using CPAP and are known to influence IOP regulation.^15^ The present study showed that 60% of patients with baseline abnormal IOP rhythms shifted to a normal 24-hour IOP profile with a nocturnal acrophase after CPAP treatment.

Our results revealed that IOP levels significantly increased in immediate response to beginning the use of CPAP. Only a few studies have attempted to evaluate the association between IOP fluctuations, OSAS and the influence of CPAP therapy on changes in IOP. In 1994, Alvarez-Sala et al.^28^ were the first to show that CPAP significantly increases IOP in patients with glaucoma. They believe that CPAP may, in fact, be counter-indicative for treating difficult-to-manage glaucoma patients. Kiekens et al.^18^ concluded that patients with OSAS demonstrated significant fluctuations in IOP over a 24-hour period, with the highest values being registered at night. They also reported that CPAP therapy was associated with an additional increase in IOP, especially at night. Cohen et al.^17^ showed that OSAS patients experience a significant rise in IOP during sleep, but that CPAP therapy did not significantly affect measured IOP values. Pepin et al.^15^ reported that treatments involving CPAP for patients with severe OSAS led to a significant increase in nocturnal IOP. Because of technical limitations, these previous studies had included noncontinuous IOP measurements, with subjects having to be awakened to conduct IOP measurements at night.

The first study that continuously evaluated IOP-related patterns in patients with OSAS was carried out by Shinmei and collaborators.^16^ They used Sensimed Triggerfish as an IOP sensor, but they did not include any patients with CPAP. They examined the impact of apnea-hypopnea events and IOP values in seven patients, reporting a significant decrease in IOP associated with apnea-hypopnea events in four of the seven patients, suggesting that negative intrathoracic pressure would cause an immediate IOP-lowering response. Interestingly, these fluctuations had no impact on the global IOP curves, which showed a similar pattern compared to those obtained in our study involving OSAS patients. As in a recent study by Carnero et al.,^29^ in which IOP was monitored using a CLS during a PSG study conducted with 20 OSAS patients without CPAP, we observed that patients with severe OSAS showed significant increases in nocturnal IOP values.

Carnero et al.^29^ showed that the length of the nocturnal IOP elevation was associated with respiratory parameters that were altered in patients with OSAS, meaning that patients who have better oxygen saturation show shorter nocturnal acrophase. Furthermore, a significant increase in IOP was observed as a response to short-term hypoxia, which returned to the baseline after hypoxia. The increase was dependent on the induced oxygen desaturation.^30^ This relationship between hypoxia and increased IOP can contribute to explain the positive correlation that has been reported between IOP levels and the severity of OSAS.^13^ CPAP therapy contribute to reduce the episodic hypoxia in OSAS; therefore we could expect a reduction in IOP. But we must take into account the effect of increased intrathoracic pressure associated with the use of CPAP on IOP. We showed that CPAP therapy was associated with a significant increase in IOP-related patterns during the initial phase of nocturnal acrophase. Despite the fact that in our study there were no statistically significant differences between the maximum and minimum IOP-related values recorded for patients before and during CPAP, we hypothesized that at the time of starting to use CPAP, IOP would probably have increased because of the sudden increase in positive intrathoracic pressure. This would have continued until it reached a plateau that would usually have finished when the patient woke up and left the bed, exhibiting a decrease in IOP that would have created a negative slope. We can therefore state that CPAP is not generally associated with significant changes in the general parameters of 24-hour monitoring of IOP. However, in a comparative assessment, we were able to detect an increase in IOP associated with starting to use CPAP therapy.

Some studies have indicated that show greater-time IOP elevations might be greater in patients with glaucoma compared to healthy controls.^31^ How these IOP fluctuations can contribute to the onset and progression of glaucoma is not completely understood, but the cumulative effects of long-term intermittent IOP elevation may result in glaucomatous damage.^32^ It is believed that the IOP increase could directly damage the optic nerve but also this elevation could affect the blood flow indirectly harming the optic nerve. Finally, it has been observed that astrocytes in the optic nerve head are mechanosensitive and can respond to the mechanical stress induced by IOP increase, reducing the trophic support or changes in extracellular remodeling in the optic nerve.^29^ The additional increase in IOP associated with CPAP use could explain the risk of glaucoma progression in certain OSAS patients despite them receiving treatment for OSAS with CPAP.^14^

The current study had some limitations. One major limitation was its relatively small sample size. As a result of this small sample size, it was not possible to make any comparisons that depended on the severity of the OSAS or on the air pressure of the CPAP or different bodily position while patients were sleeping. Despite these limitations, this study is the first one that has shown the IOP effect of CPAP on sleep, as measured by continuous monitoring under physiological conditions. Further research will be needed to define the OSAS parameters and CPAP parameters that affect IOP-related measuring by CLS.

In conclusion, the present study identified that the majority of patients with severe OSAS had a circadian fluctuation of their IOP-related pattern with a nocturnal IOP increase that was probably related to their supine position. CPAP therapy was associated with an additional and significant increase in IOP-related values that mainly occurred one hour after starting CPAP use. We should therefore take this CPAP effect into account in OSAS patients with glaucoma, particularly in the case of a progression in the glaucomatous disease despite the IOP values being well controlled.
